# Two Compartment Evaluation of Liver Grafts During Acellular Room Temperature Machine Perfusion (acRTMP) in a Rat Liver Transplant Model

**DOI:** 10.3389/fmed.2022.804834

**Published:** 2022-02-24

**Authors:** Nader Abraham, Min Zhang, Paul Cray, Qimeng Gao, Kannan P. Samy, Ryan Neill, Greta Cywinska, JonCarlo Migaly, Riley Kahan, Arya Pontula, Samantha E. Halpern, Caroline Rush, Jude Penaflor, Samuel J. Kesseli, Madison Krischak, Mingqing Song, Matthew G. Hartwig, Justin J. Pollara, Andrew S. Barbas

**Affiliations:** Duke Ex-Vivo Organ Lab (DEVOL) – Division of Abdominal Transplant Surgery, Duke University, Durham, NC, United States

**Keywords:** liver transplantation, subnormothermic machine perfusion, isogeneic rat liver graft, ischemia—reperfusion, graft evaluation, bile analysis

## Abstract

**Background:**

Subnormothermic machine perfusion (SNMP) of liver grafts is currently less clinically developed than normothermic and hypothermic approaches, but may have logistical advantages. At intermediate temperatures, the oxygen demand of the graft is low enough to be satisfied with an acellular perfusate, obviating the need for oxygen carrying molecules. This intermediate metabolic rate, however, is sufficient to support the production of bile, which is emerging as an important indicator of graft injury and viability. In this study, we hypothesized that the biliary compartment would be more sensitive than perfusate in detecting graft injury during SNMP.

**Methods:**

To test this hypothesis in a rat model, we performed liver transplants with DCD and control liver grafts after 1 h of acellular room temperature machine perfusion (acRTMP) or static cold storage (SCS). Point of care liver function tests were measured in biliary and perfusate samples after 1 h of machine perfusion. Following transplantation, rats were sacrificed at 24 h for assessment of post-transplant graft function and histology.

**Results:**

All point-of-care liver function tests were significantly more concentrated in the biliary compartment than the perfusate compartment during acRTMP. DCD liver grafts could be distinguished from control liver grafts by significantly higher markers of hepatocyte injury (AST, ALT) in the biliary compartment, but not in the perfusate compartment. Classical markers of cholangiocyte injury, such as gammy-glut amyl transferase (GGT), amylase (AML), and alkaline phosphatase were detectable in the biliary compartment, but not in the perfusate compartment. In comparison to SCS, graft preservation by acRTMP produced a significant survival benefit in DCD liver transplantation (75 vs. 0%, *p* < 0.0030).

**Conclusion:**

Together, these findings demonstrate that during acRTMP, the biliary compartment may be a more sensitive indicator of graft injury than the perfusate compartment. Moreover, acRTMP provides superior graft preservation to SCS in rat DCD liver transplantation.

## Introduction

*Ex vivo* machine perfusion has emerged as a superior method for graft preservation over static cold storage (SCS) in liver transplantation (1, 2). However, consensus has yet to be achieved regarding the optimal conditions for machine perfusion of liver grafts. Both hypothermic (4–10°C) (1) and normothermic (33–37°C) (2) machine perfusion has demonstrated benefit over SCS in clinical trials, but also come with specific limitations. Under hypothermic conditions, reduced graft metabolic activity limits the ability to perform viability testing of marginal liver grafts. In contrast, under normothermic conditions, the high oxygen demand of metabolically active grafts requires a more complex perfusion system and a perfusate that includes oxygen carriers. Alternatively, perfusion at an intermediate temperature range (22–30°C), termed subnormothermic machine perfusion (SNMP), has some potential advantages that warrant further exploration (3). At intermediate temperatures, the oxygen requirement of the graft is low enough to be met with a simple acellular perfusate, yet the metabolic activity of the graft is sufficiently high to support the production of bile, which is emerging as an important indicator of graft injury and viability (4–7).

In order to use machine perfusion as a platform to assess the viability of marginal liver grafts, there is an urgent need to define sensitive and specific parameters in perfusate and bile, ideally using point-of-care tests. Thus far, more investigation has focused on the perfusate compartment, with several analytes of interest emerging as potentially useful. Among these, lactate clearance appears most promising, and has been included in normothermic machine perfusion protocols as a major criterion defining graft viability (8, 9). However, due to the heterogeneity of machine perfusion protocols, including variations in perfusate composition and volume, standardization of perfusate analysis may prove challenging. In contrast, interrogation of the biliary compartment during machine perfusion may provide a more standardized method of comparison. Thus far, biliary compartment evaluation has focused on a limited number of parameters indicative of cholangiocyte function, namely biliary pH and biliary glucose levels. Alkaline biliary pH and low biliary glucose levels have been correlated with a reduced risk of ischemic cholangiopathy in DCD liver transplants (4). Further development of approaches to evaluate the biliary compartment are necessary to advance the field.

In this study, we sought to perform a more comprehensive assessment of the biliary compartment during machine perfusion of liver grafts in a subnormothermic model. We hypothesized that the biliary compartment would be more sensitive than the perfusate compartment for the detection of liver graft injury using standard (10) point-of-care tests. We tested this hypothesis in a rat model with DCD and control liver grafts undergoing acellular room temperature machine perfusion (acRTMP), followed by liver transplantation. We demonstrate that markers of graft injury are all significantly more concentrated in the biliary compartment relative to perfusate, and that differences between DCD and control livers can only be distinguished by examination of the biliary compartment. Moreover, we demonstrate that acRTMP rescues a uniformly lethal DCD injury in our rat model, resulting in a significant increase in post-transplant survival compared to SCS.

## Methods

### Laboratory Animals

**Figure 1:**
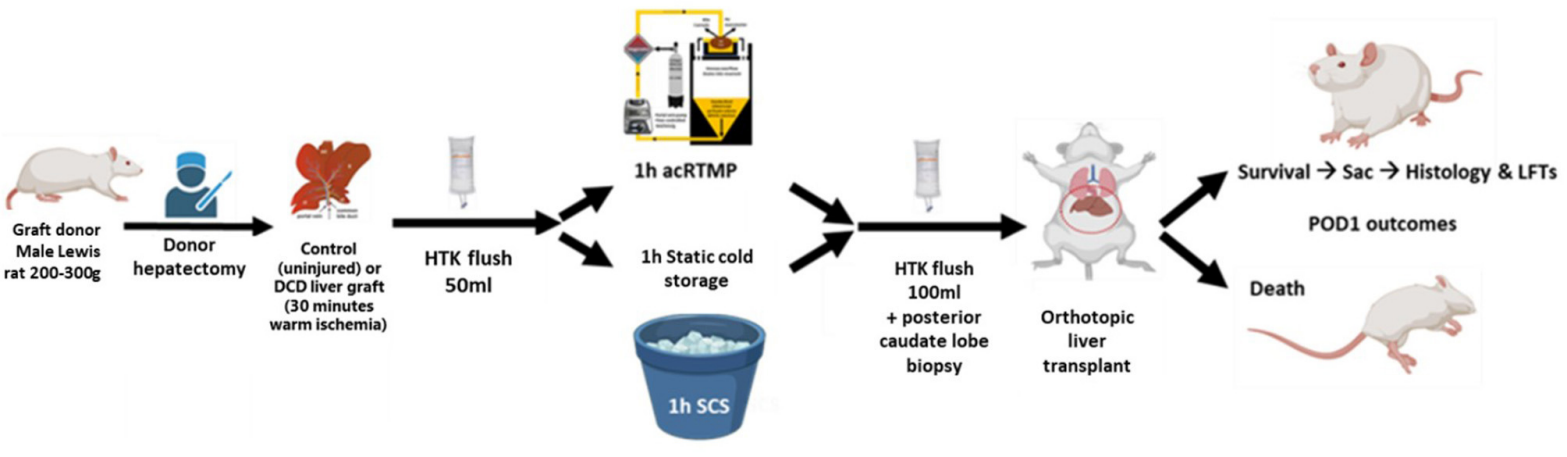
Experimental design. Control and DCD liver grafts were recovered and preserved by 1 h of acellular room temperature machine perfusion, followed by orthotopic liver transplantation. Comparisons were made to DCD liver grafts preserved by static cold storage.

Inbred male Lewis rats weighing 250–300 g (Charles River Laboratories, Boston, MA, USA) were used as both graft donors and recipients for syngeneic orthotopic liver transplantation. The animals were housed in accordance with National Research Council guidelines, and the experimental protocols were approved by the institutional animal care and use committee at Duke University (Durham, NC, USA). The animals were divided into three groups: the DCD30 + 1 h acRTMP group (*n* = 8, 30 min WIT, 1 h acRTMP, and subsequent transplantation), the Control + 1 h acRTMP group (*n* = 8, 0 min WIT, 1 h acRTMP, and transplantation), the DCD30 + 1 h SCS group (*n* = 6, 30 min WIT, 1 h of SCS in HTK solution, and transplantation). Experimental protocol is indicated in Figure 1.

### Liver Procurement

Animals were weighed and anesthesia was induced with 5% isoflurane (Baxter, Deerfield, IL, USA) for 5 min at 2 L/min of 100% Oxygen (Airgas, Durham, NC). The abdominal cavity was accessed via a cruciate incision, then anesthesia was maintained at 0.5–1% isoflurane for the duration of the procedure. The left phrenic vein was ligated and divided between 7 and 0 Silk ties. The liver was freed of its surrounding ligaments by sharp dissection. The infrahepatic vena cava (IHVC) was mobilized by blunt dissection and elongated by ligation and dissection of the right adrenal vein, the posterior lumbar plexus, and right renal vein. The bile duct was cannulated (SURFLO 24-gauge polyethylene stent; Terumo Medical Corp, Somerset, NJ, USA) and bluntly dissected. The portal vein (PV) was mobilized, and its most proximal side vessels (gastroduodenal and splenic veins) were ligated and divided between 7 and 0 Silk ties. The hepatic artery was ligated and divided. In the DCD30 groups, The PV was cross clamped, and a sternotomy was performed to induce bilateral lung collapse, marking the start of ischemic time, for a total of 30 min. The grafts were flushed with 50 ml of ice-cold HTK solution (Custodiol; Essential pharmaceuticals, LLC, Durham, NC, USA), followed by graft excision.

### Acellular Room Temperature Machine Perfusion (acRTMP)

**Figure 2:**
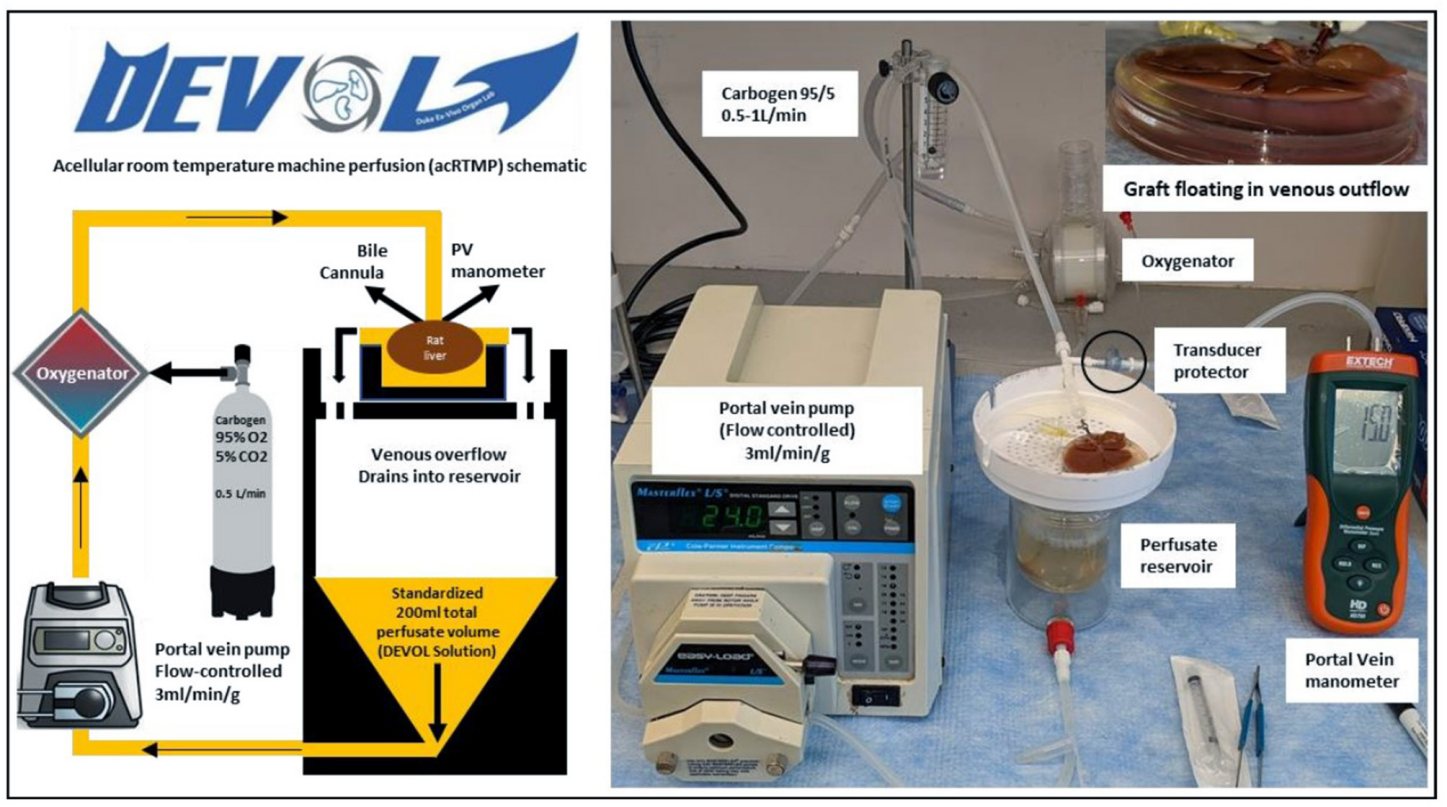
Acellular room temperature machine perfusion schematic. Control and DCD liver grafts were perfused continuously at room temperature for 1 h using an acellular perfusate with physiologic pH, electrolyte composition, and oncotic pressure.

Machine perfusion was performed in a circuit that consisted of a perfusate reservoir situated underneath a graft chamber, a peristaltic pump, and a membrane oxygenator (Figure 2). The liver was perfused through an 18-gauge metal cannula (Harvard Apparatus, Boston, MA) that was connected to the PV. Temperature within the system was left to equilibrate passively with the ambient room temperature and measured between 21 and 25°C. The total perfusate volume was 200 ml and consisted of an acellular perfusate consisting of human albumin and an electrolyte mixture which recapitulates the pH balance, oncotic, and osmotic pressures of human plasma. The perfusate was supplemented with piperacillin/tazobactam (2.25 g/L) (Sandoz, Princeton, NJ, USA), dexamethasone (100 mg/L) (Mylan, Greensboro, NC, USA), and heparin (10,000 U/L). The membrane oxygenator (Capiox FX05, Elkton, MD, USA) used carbogen (95% O_2_ and 5% CO_2_) to oxygenate the perfusate and ensure a minimum inflow PO_2_ >500 mmHg. Portal vein flow rate was set at 3 ml/min/g liver tissue. Portal vein pressure was continuously monitored using a digital differential pressure manometer (HD750, Extech, Nashua, NH, USA), connected directly to the portal venous line via a transducer protector (MPC-85TE, MPC, Harlan, IA, USA). At the end of machine perfusion, grafts were cooled down again with an ice-cold 100-ml HTK flush at a maximum of 15 mmHg through the portal vein, then transferred back to the surgery room packaged in ice-cold HTK.

### Perfusate Analysis

Perfusate samples were collected before the liver was connected to the circuit (T0h) and at the end of the perfusion (T1h). A single bile sample consisting of the entire bile volume that was produced during the 1 h acRTMP was collected at the end of perfusion and its volume was quantified via reverse pipetting. Both perfusate and bile samples were tested for glucose level using One touch Verio flex™ handheld glucometer (Lifescan IP holdings, LLC, Malvern, PA, USA), lactate level using Lactate plus™ handheld (Nova Biomedical, Waltham, MA, USA), and liver function panel using Piccolo Xpress™ chemistry analyzer (Abaxis, Union City, CA, USA).

### Graft Back-Table Preparation, Biopsy, and Orthotopic Liver Transplantation

Upon the end of the preservation period, the portal vein cannula was removed and the portal vein and infrahepatic inferior vena cava (IHIVC) were cuffed using cuffs fashioned from 16-gauge PTFE extruded plastic tubing (Zeus, Rangeburg, SC, USA) and 14-gauge angiocath (BD, Franklin lakes, NJ, USA), respectively, as described by Delrivière et al. (11). The suprahepatic vena cava (SHVC) was tailored for a sutured anastomosis. The pedicled posterior caudate lobe (PCL) was then resected, to serve as a post-preservation biopsy, and fixed in 10% neutral-buffered Formalin (NBF). Biopsy site hemostasis was achieved by tying the PCL lobe vascular pedicle using 5-0 silk ties, and surface electrocautery. After back-table preparation of the graft, the recipient hepatectomy was performed. After hepatectomy, graft implant started with suture anastomosis of the supra-hepatic inferior vena cava (SHIVC) using 8-0 Nylon suture (AROSurgical instruments corporation, Inc., Newport beach, California, USA). Portal cuff anastomosis was performed afterwards, followed by graft reperfusion. The anhepatic time was maintained below 20 min. Graft reperfusion was followed by anastomoses of the IHIVC and bile duct. The hepatic artery was not re-anastomosed. Before closure, the abdominal cavity was irrigated with lactated ringer's solution, and dried with sterile gauze. The anterior abdominal wall and the skin were then closed. All recipients received a subcutaneous injection of 5-ml of lactated ringer's solution for volume resuscitation, baytril as an antibiotic and buprenorphine for pain control. The rat was then placed in a chamber with continuous 100% O_2_ flow at 3–4 L/min, on a heating pad, for 1-h to recover.

### Post-operative Day 1 (POD1) Endpoint, Sacrifice, and Analysis

On POD1, the recipients, if found alive, were re-anesthetized and the abdominal incision re-opened. A blood sample was collected from the IHIVC into EDTA anticoagulation tubes, and liver sections were resected and fixed in 10% NBF. The blood samples were analyzed for glucose level using One touch Verio flex™ handheld glucometer (Lifescan IP holdings, LLC, Malvern, PA, USA), lactate level using Lactate plus™ handheld (Nova Biomedical, Waltham, MA, USA), and Liver function panel using Piccolo Xpress™ chemistry analyzer (Abaxis, Union City, CA, USA). Liver tissue was embedded in paraffin, processed, and stained with H&E for reading of the Suzuki injury scores (12) by a blinded pathologist (Table 1).

**Table 1:** Suzuki score for the assessment of liver damage following hepatic ischemia/reperfusion.

**Score**	**Congestion**	**Vacuolization**	**Necrosis**
0	None	None	None
1	Minimal	Minimal	Single cell necrosis
2	Mild	Mild	<30%
3	Moderate	Moderate	<60%
4	Severe	Severe	>60%

### Statistical Analysis

A paired Student's *t*-test was applied to compare values for each nominal parameter independently. One-way ANOVA was applied to compare ordinal values. The results were deemed statistically significant if time point comparisons had a *P*-value <0.05. Statistical significance is indicated in each figure where applicable. Figures were created using Graphpad prism, Microsoft Powerpoint, and Biorender.com.

## Results

### Perfusion Vascular Parameters

**Figure 3:**
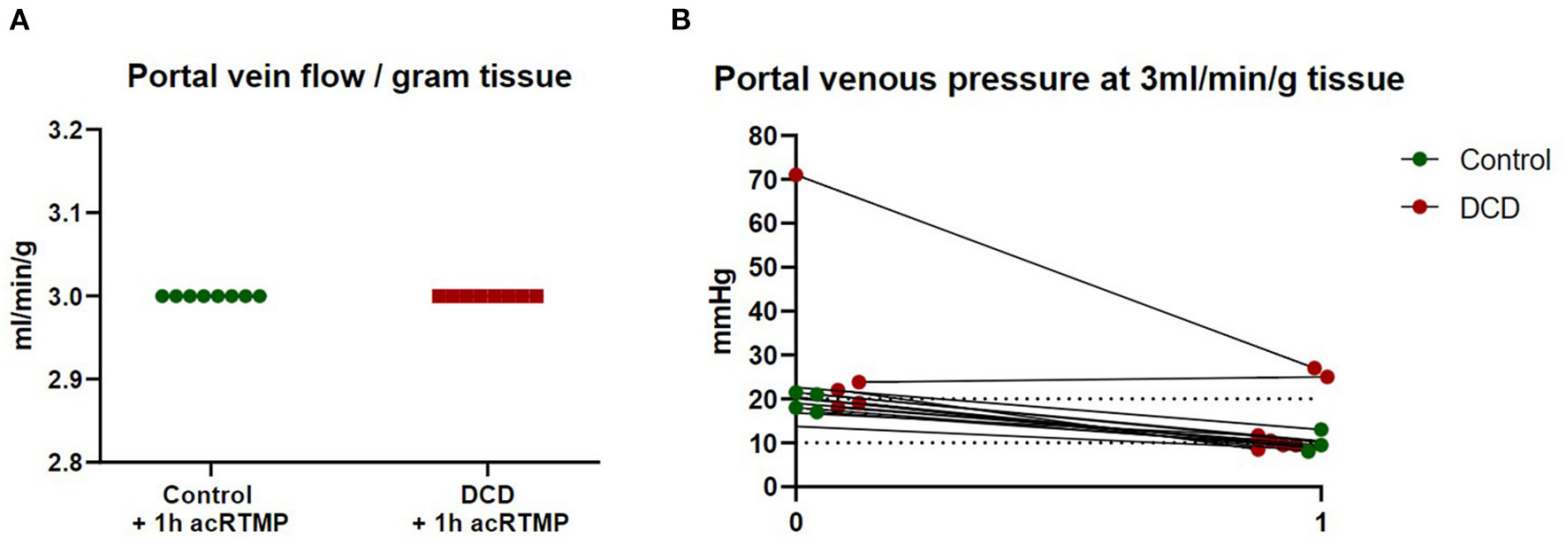
Portal vein flow and pressure. **(A)** Liver grafts were perfused with fixed flow rates of 3 ml/gram tissue. **(B)** Portal vein pressure was measured continuously during perfusion.

Both control and DCD liver grafts were maintained at a fixed portal vein flow rate of 3 ml/min/gram tissue (Figure 3). Portal vein pressure was measured continuously and demonstrated no significant differences between control and DCD liver grafts at time 0 or 1 h.

### Two-Compartment Evaluation of Graft Injury: Perfusate vs. Bile

**Figure 4:**
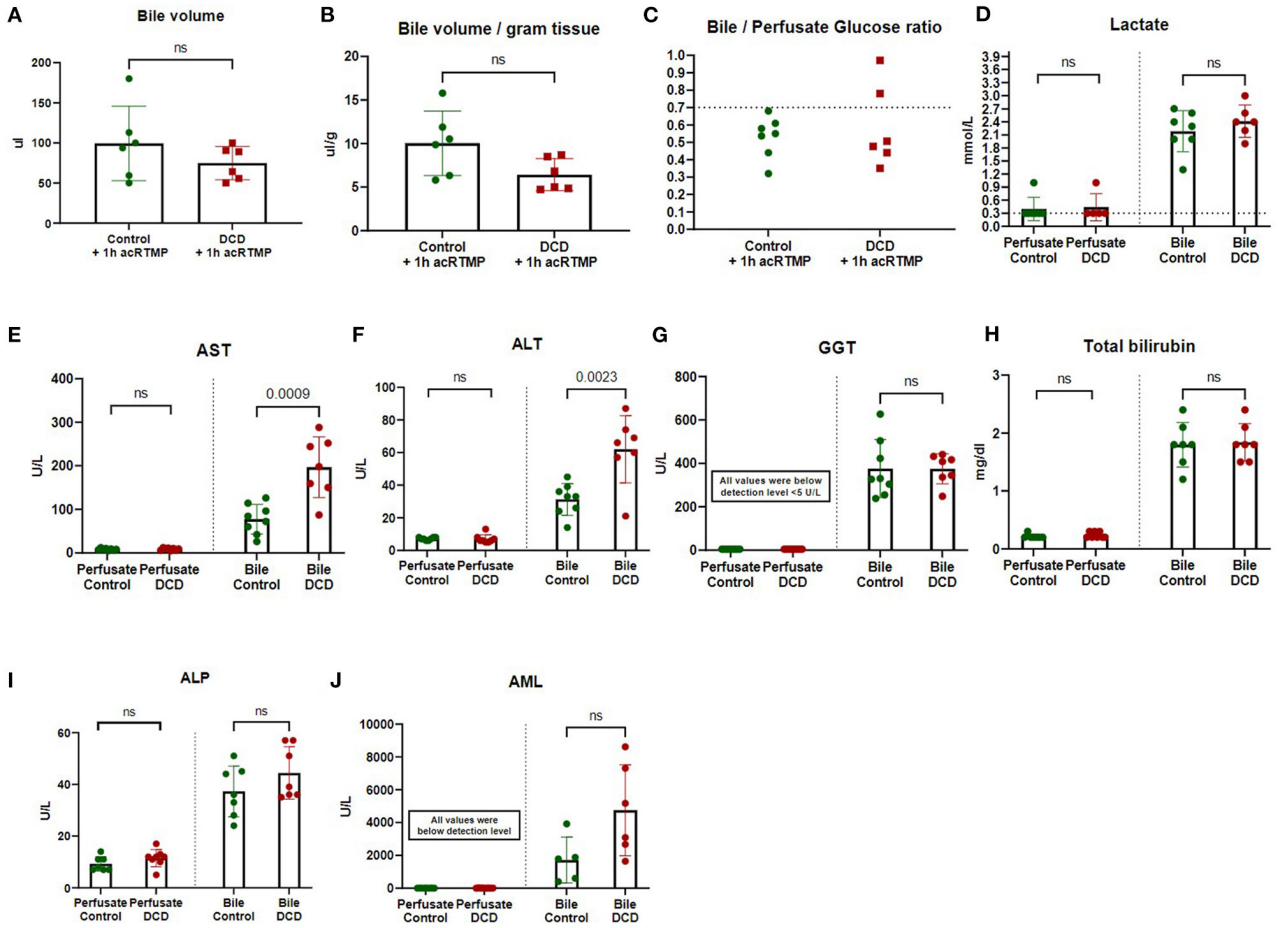
**(A-J)** Two-compartment evaluation for liver graft assessment during machine perfusion. Biochemical parameters were measured and compared in biliary vs. perfusate compartments.

Bile volume production during 1 h of acRTMP was similar between control and DCD grafts (Figures 4A,B). Bile/perfusate glucose ratio was also similar between control and DCD grafts (Figure 4C). Several point-of-care markers of graft injury (lactate, AST, ALT, GGT, Tbili, ALP, and AML) were measured and compared in both perfusate and biliary compartments. For all parameters, the concentration in the perfusate compartment was significantly lower than the biliary compartment (Figures 4D–J). Moreover, there were no significant differences that could distinguish control vs. DCD liver grafts based on any parameter measured in the perfusate compartment, including lactate, AST, and ALT. In contrast, in the biliary compartment, DCD grafts demonstrated significantly elevated AST and ALT in comparison to control grafts.

### Post-preservation Histology

**Figure 5:**
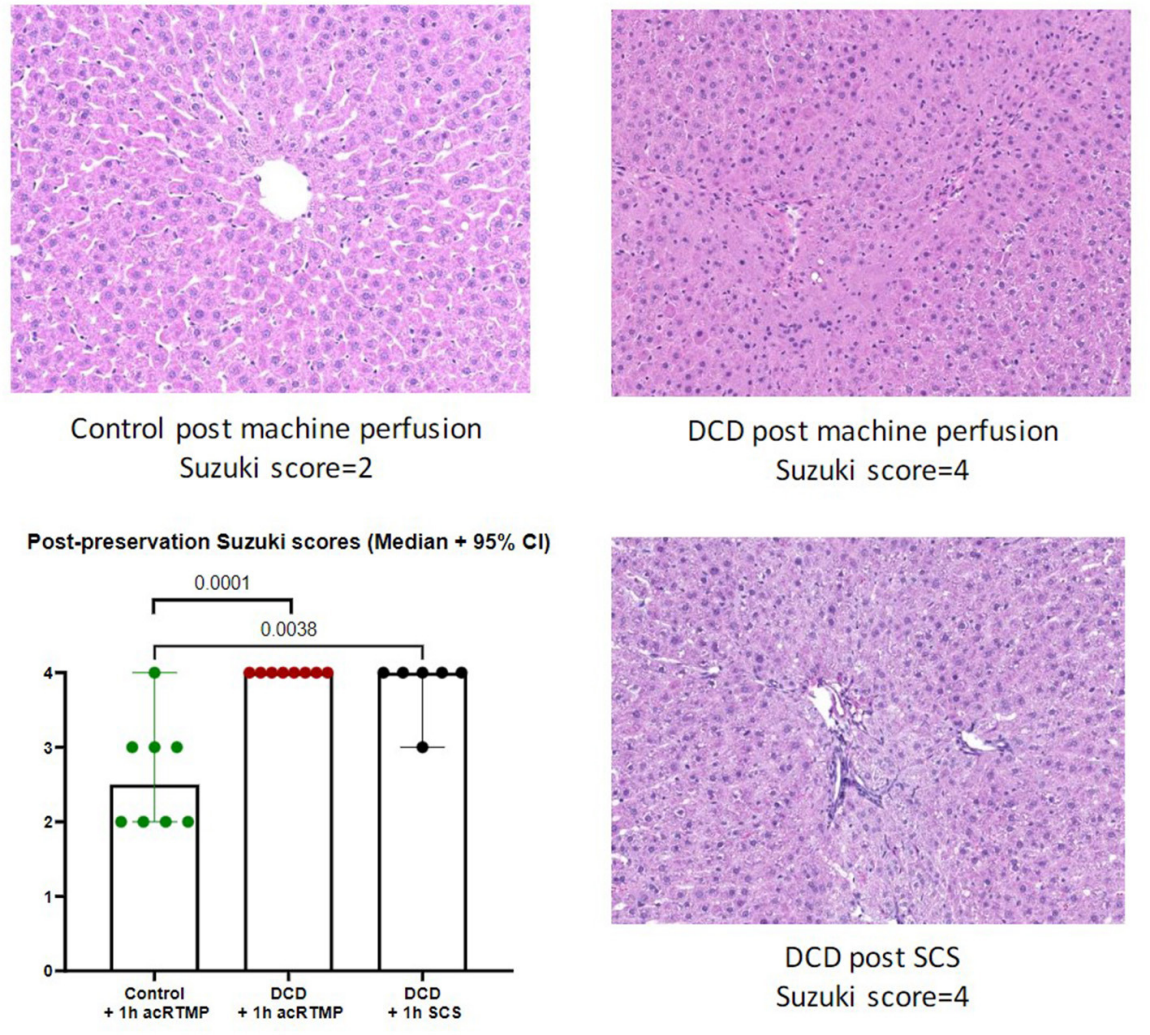
Graft histologic injury by Suzuki score following preservation by SCS vs. acRTMP.

**Figure 6:**
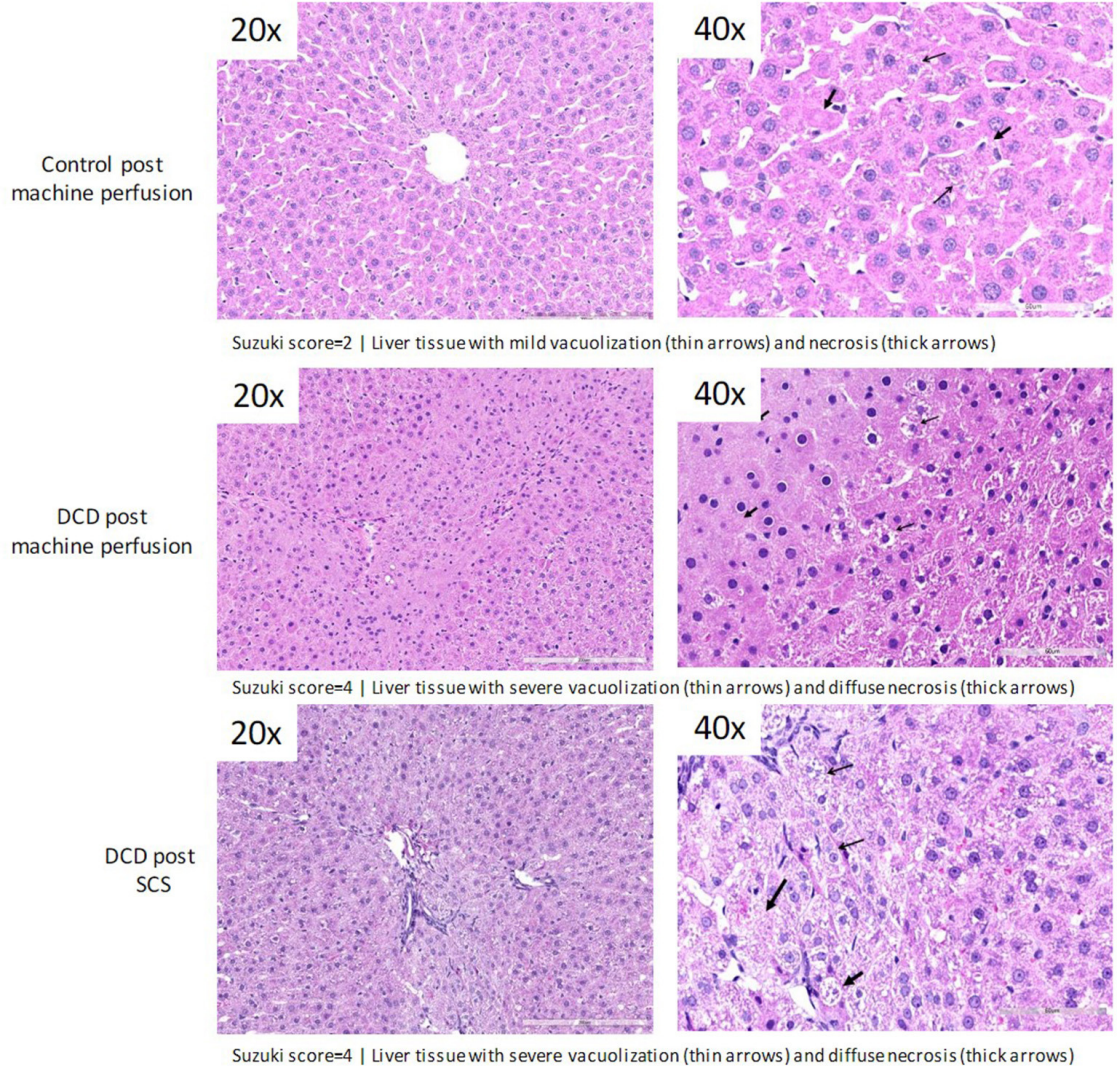
Higher magnification of graft histologic injury by Suzuki score following preservation by SCS vs. acRTMP.

Following 1 h of preservation of liver grafts by either acRTMP or SCS, the caudate lobe was biopsied and processed for histological examination. DCD grafts, preserved by either SCS or acRTMP, demonstrated significant injury, with most grafts demonstrating a Suzuki score of 4 (Figures 5, 6). In contrast, control grafts with no warm ischemic injury and preserved by acRTMP demonstrated significantly lower Suzuki scores.

### Liver Transplant Survival

**Figure 7:**
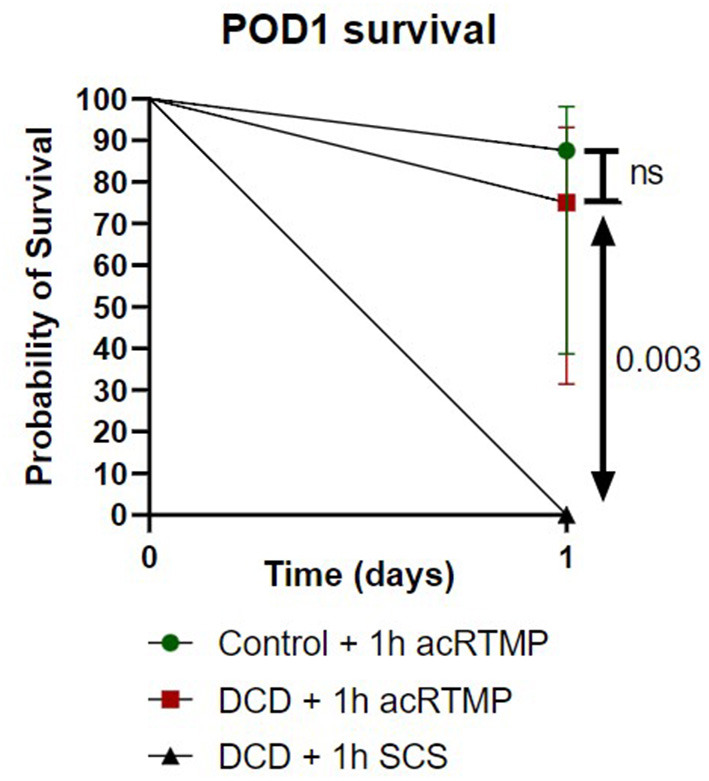
Liver transplant survival to study end-point.

Survival to the post-operative day 1 study endpoint was compared for all groups (Figure 7). Liver transplantation with DCD grafts preserved by SCS was uniformly fatal, with no survivors. Most deaths in the SCS group occurred within 4 h, suggestive of primary graft non-function. In contrast, recipients of DCD liver grafts preserved by acRTMP demonstrated significantly higher survival, with 6/8 (75%) surviving to the study endpoint. Recipients of control liver grafts demonstrated the best survival, with 7/8 (87.5%) surviving to the study endpoint, although this was not significantly higher than the DCD group.

### Graft Function on Post-operative Day 1

**Figure 8:**
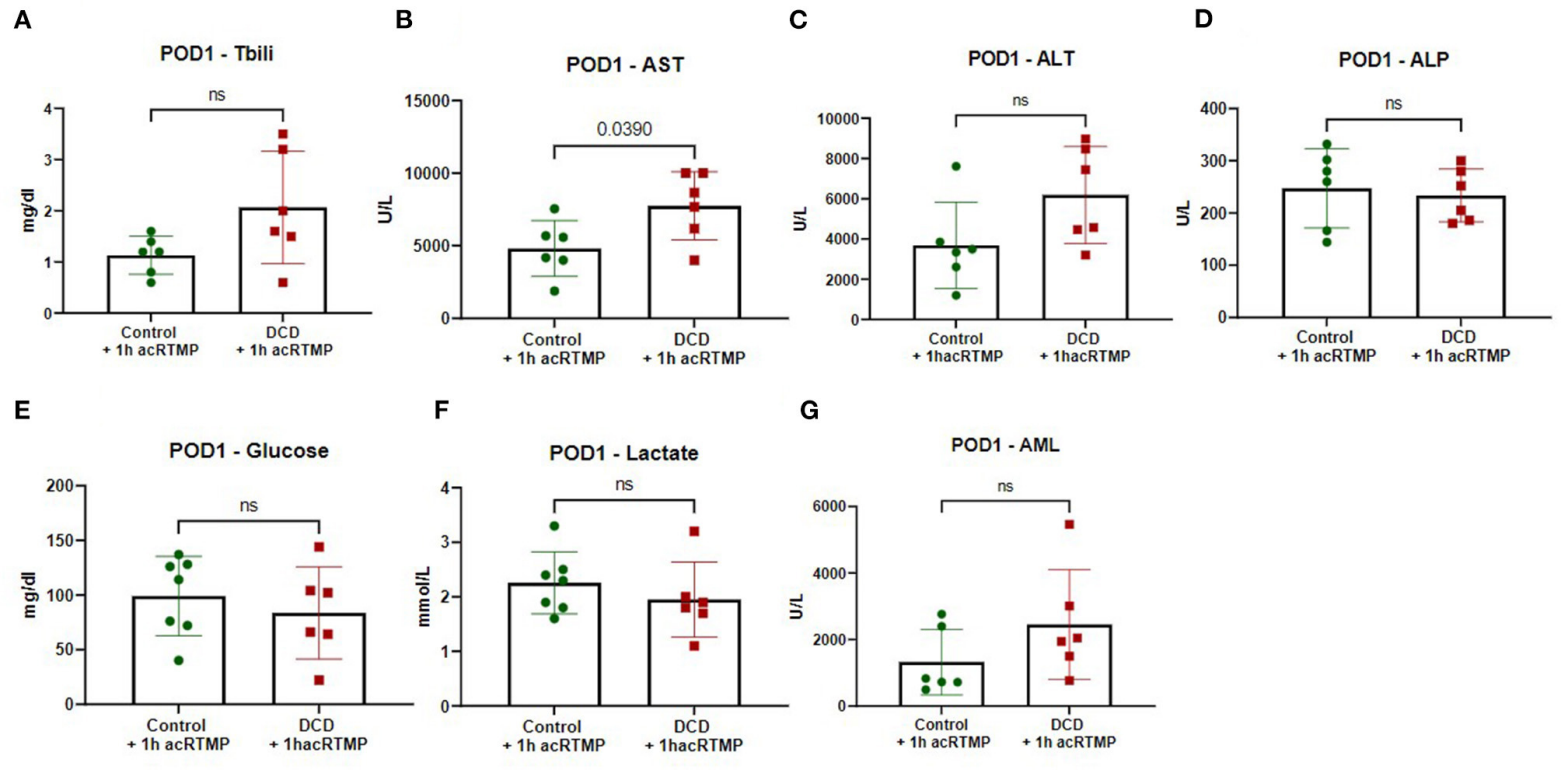
**(A-G)** Biochemical parameters in recipients on post-operative day 1 following liver transplant.

For animals who survived to the post-operative day 1 study endpoint, several markers of graft injury and function were measured in the blood. AST and ALT were significantly higher in the DCD group in comparison to control, but there were no differences in any of the other measured parameters (Figure 8).

### Post-transplant Histology

**Figure 9:**
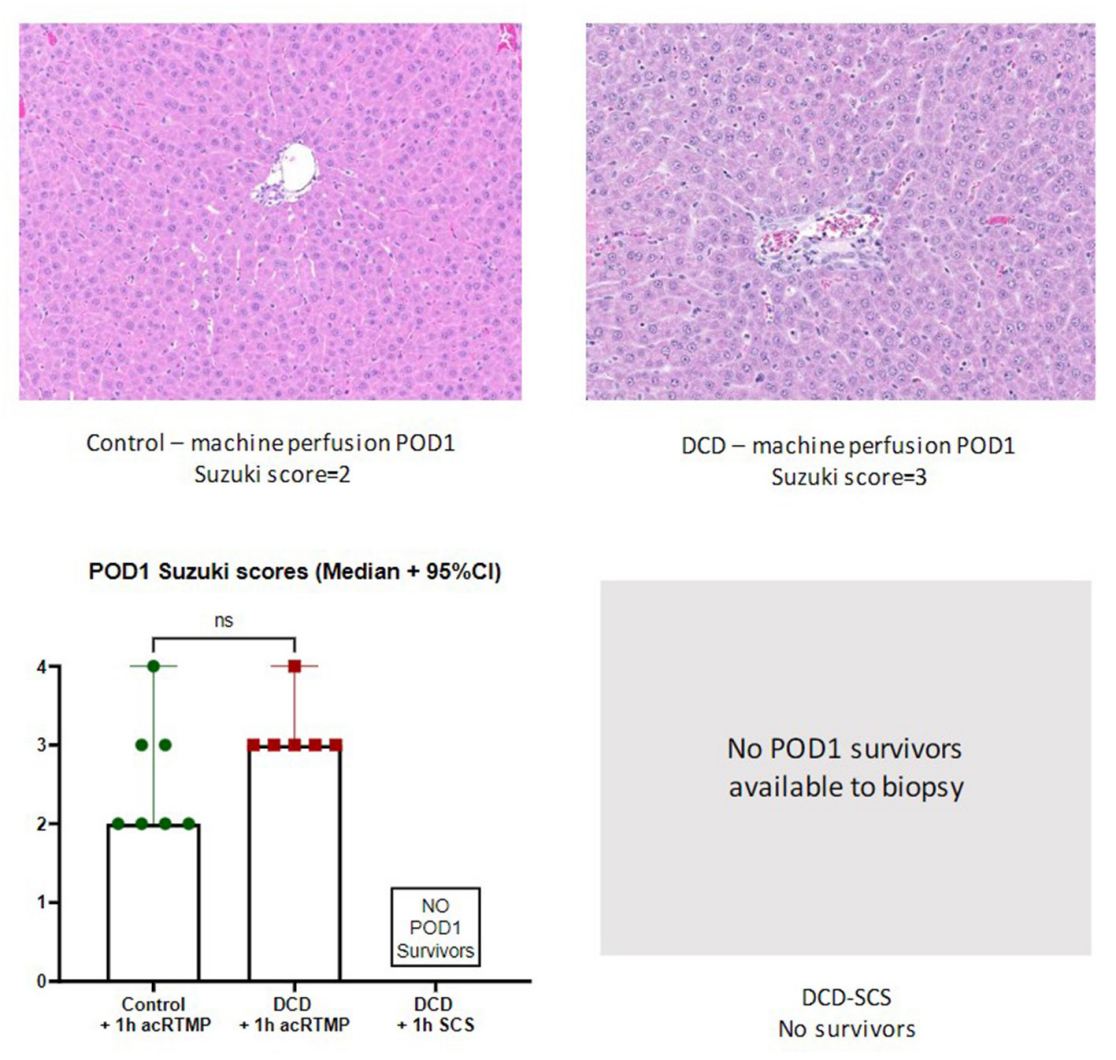
Graft histologic injury by Suzuki score at study end-point.

**Figure 10:**
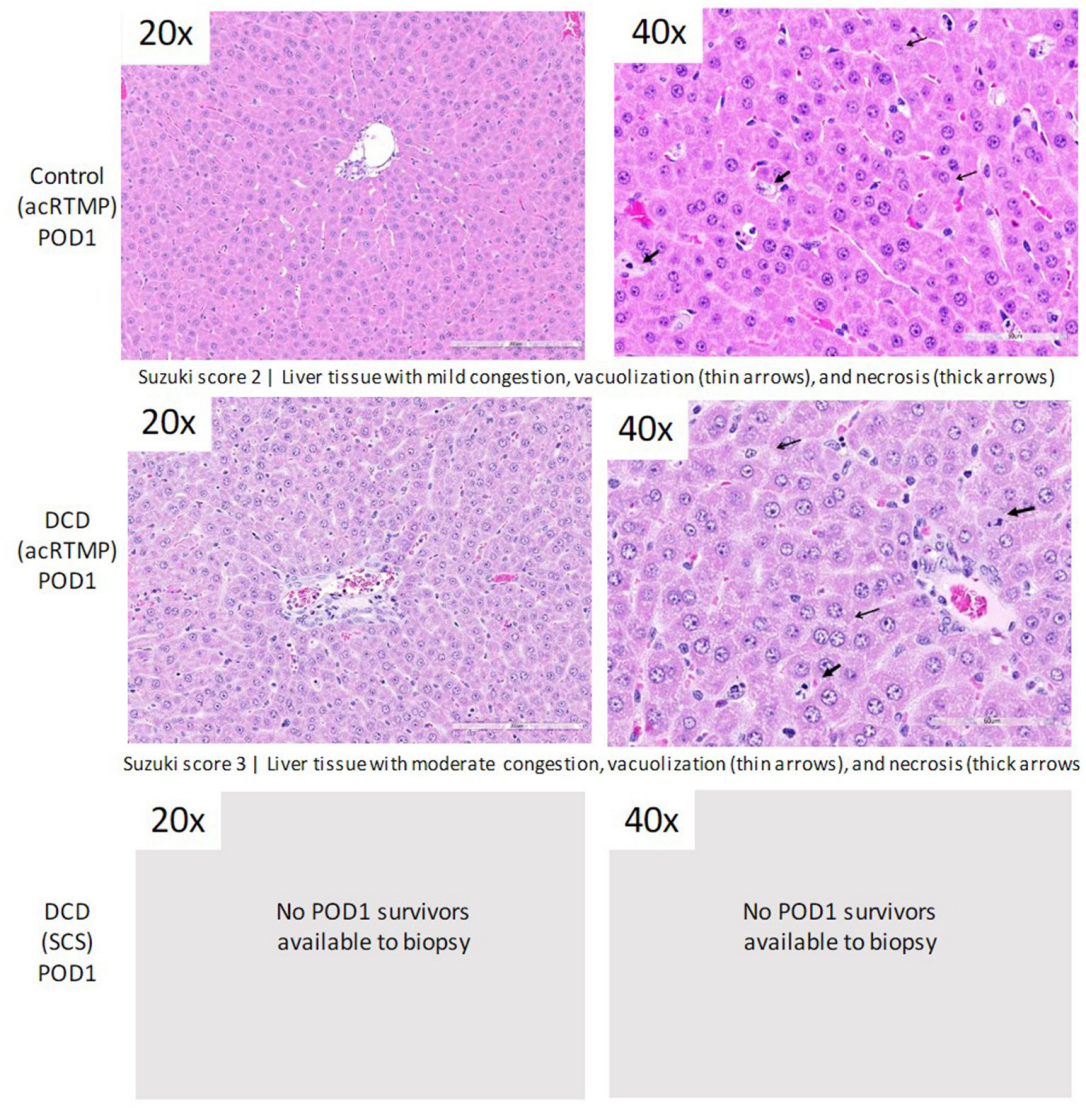
Higher magnification of graft histologic injury by Suzuki score at study end-point.

Liver grafts were biopsied and processed for histologic examination at the study endpoint. There were no significant differences in graft level of injury by Suzuki score between surviving control and DCD liver grafts, all which had been preserved by acRTMP (Figures 9, 10).

## Discussion

In this study, using a rat model of subnormothermic liver perfusion followed by transplantation, we demonstrate two primary findings: (1) machine perfusion produces a significant survival benefit over SCS for DCD liver grafts, and (2) during machine perfusion, the biliary compartment appears to be more sensitive than the perfusate compartment for the detection of liver graft injury.

Our findings demonstrating the potential benefits of subnormothermic machine perfusion are consistent with recent literature (3, 13–23), although the vast majority of previous studies did not include liver transplantation to assess graft function and recipient survival. Among these studies, the only one that included transplantation of rat liver grafts was performed by Tolboom et al. (16). In this study, DCD liver grafts were preserved by subnormothermic perfusion (at 20 and 30°C) using a more complex perfusate containing RBCs and a circuit incorporating continuous dialysis. They similarly demonstrated a 100% mortality in DCD liver grafts preserved by SCS, with significant survival benefit seen with subnormothermic perfusion.

Regardless of the approach used for machine perfusion of liver grafts (hypothermic, subnormothermic, or normothermic), one of the key issues that remains under investigation is how to best determine graft viability during the perfusion period. Most studies thus far have focused on analytes in the perfusate compartment, including markers of hepatocyte injury (5, 10, 24–27) and perfusate lactate (8, 9). However, due to the heterogeneity of perfusate volume and composition across platforms, it is difficult to make generalizable comparisons with defined cut-offs for perfusate analytes. In contrast, analysis of the biliary compartment may represent a more standardized approach. Thus far, biliary pH and glucose have been the primary parameters of interest in the bile (7) and have been correlated with clinical outcomes in human liver transplantation (4). Our study expands on this concept by demonstrating that classical markers of graft injury are all more concentrated in the biliary compartment compared to the perfusate. Moreover, in this study DCD grafts could only be differentiated from uninjured control grafts by biliary AST and ALT levels, while perfusate levels showed no difference between groups.

There are some limitations of this study that should be discussed, primarily related to study design. Since our primary interests involved graft assessment during machine perfusion and early graft function following transplantation, we chose a study endpoint of 24 h and used a syngeneic transplant model. As such, we are unable to comment on longer-term graft function, recipient survival, or the effects of alloimmunity. With regard to perfusion duration, we selected a relatively short perfusion time of 1 h in this study based on the literature that has been established for hypothermic oxygenated perfusion, in which clinical benefit has been established using 1–2 h perfusion times (1, 28). This perfusion time is convenient and practical as it can be applied to liver grafts as the recipient operation commences. Similar to the findings in hypothermic oxygenated perfusion, we were able to demonstrate a clinical benefit for DCD liver grafts with just 1 h of perfusion. Finally, we used a flow-controlled perfusion approach, rather than pressure-controlled, which has potential to lead to sinusoidal endothelial injury if the liver graft is subjected to high pressure states. However, most grafts in the study demonstrated physiologic portal vein pressure measurements by the end of 1 h of perfusion.

In conclusion, subnormothermic perfusion represents a promising approach to liver graft preservation, combining simple logistics while retaining the ability to perform graft assessment. A more comprehensive analysis of the biliary compartment to include markers of graft injury may help refine assessment of graft viability.

## Data Availability Statement

The original contributions presented in the study are included in the article/supplementary material, further inquiries can be directed to the corresponding author.

## Ethics Statement

The animal study was reviewed and approved by Duke Institutional Animal Care and Use Committee.

## Author Contributions

AB, NA, and MZ conceived and planned the experiments. NA, MZ, RN, GC, JM, AP, SK, and MK carried out the experiments. AB took the lead in writing the manuscript, with help from NA. All authors contributed to the interpretation of the results. All authors provided critical feedback and helped shape the research, analysis, and manuscript. All authors contributed to the article and approved the submitted version.

## Funding

This work was funded by NIH K08 AI150990 to AB and NIH R01 AI153274 to JP and AB.

## Conflict of Interest

The authors declare that the research was conducted in the absence of any commercial or financial relationships that could be construed as a potential conflict ofinterest.

## Publisher's Note

All claims expressed in this article are solely those of the authors and do not necessarily represent those of their affiliated organizations, or those of the publisher, the editors and the reviewers. Any product that may be evaluated in this article, or claim that may be made by its manufacturer, is not guaranteed or endorsed by the publisher.
